# Photoallergic reaction in a patient receiving vandetanib for metastatic follicular thyroid carcinoma: a case report

**DOI:** 10.1186/s12895-015-0022-1

**Published:** 2015-02-13

**Authors:** Jennifer Goldstein, Anisha B. Patel, Jonathan L. Curry, Vivek Subbiah, Sarina Piha-Paul

**Affiliations:** Division of Cancer Medicine, Medical Oncology and Hematology Fellowship Program, University of Texas MD Anderson Cancer Center, 1515 Holcombe Boulevard, Houston, TX 77030 USA; Department of Dermatology, University of Texas MD Anderson Cancer Center, 1515 Holcombe Boulevard, Houston, TX 77030 USA; Department of Pathology, University of Texas MD Anderson Cancer Center, 1515 Holcombe Boulevard, Houston, TX 77030 USA; Department of Investigational Cancer Therapeutics, University of Texas MD Anderson Cancer Center, 1515 Holcombe Boulevard, Unit 455, Houston, TX 77030 USA

**Keywords:** Photoallergic reaction, Vandetanib, Metastatic follicular thyroid carcinoma, Tyrosine kinase inhibitor, Investigational cancer therapeutics

## Abstract

**Background:**

Novel targeted agents have been increasingly developed and tested in clinical trials over the past 5–10 years, many with unknown and unanticipated side effects. We describe here a case of a patient with a history of metastatic follicular thyroid carcinoma that we believe developed vandetanib–associated photoallergic dermatitis while enrolled on a phase 1 clinical trial.

**Case presentation:**

A 51-year-old Caucasian female with poorly differentiated, metastatic follicular thyroid carcinoma presented with a cutaneous eruption that developed over 3 to 4 days while treated on phase 1 clinical trial with vandetanib-based therapy. Given the concern for photoallergic dermatitis, vandetanib was discontinued and supportive care provided including topical and oral steroid administration. Her cutaneous eruption improved and she was successfully re-challenged with vandetanib.

**Conclusion:**

Tyrosine kinase inhibitors, such as typo-vandetinib, with various therapeutic targets have come to the forefront of oncologic therapy in recent years. It is important to have a better understanding of the side effect profile and management in order to anticipate adverse events and maintain patient safety in future clinical trials.

## Background

Novel targeted agents have been increasingly developed and tested in clinical trials over the past 5–10 years. In clinical trials, drug reactions cannot always be anticipated and novel side effects can be encountered. Vandetinib is a multikinase inhibitor. Herein, we describe a patient with metastatic follicular thyroid carcinoma who we believe developed vandetanib–associated photoallergic dermatitis while enrolled on a phase 1 clinical trial.

## Case presentation

**Figure 1:**
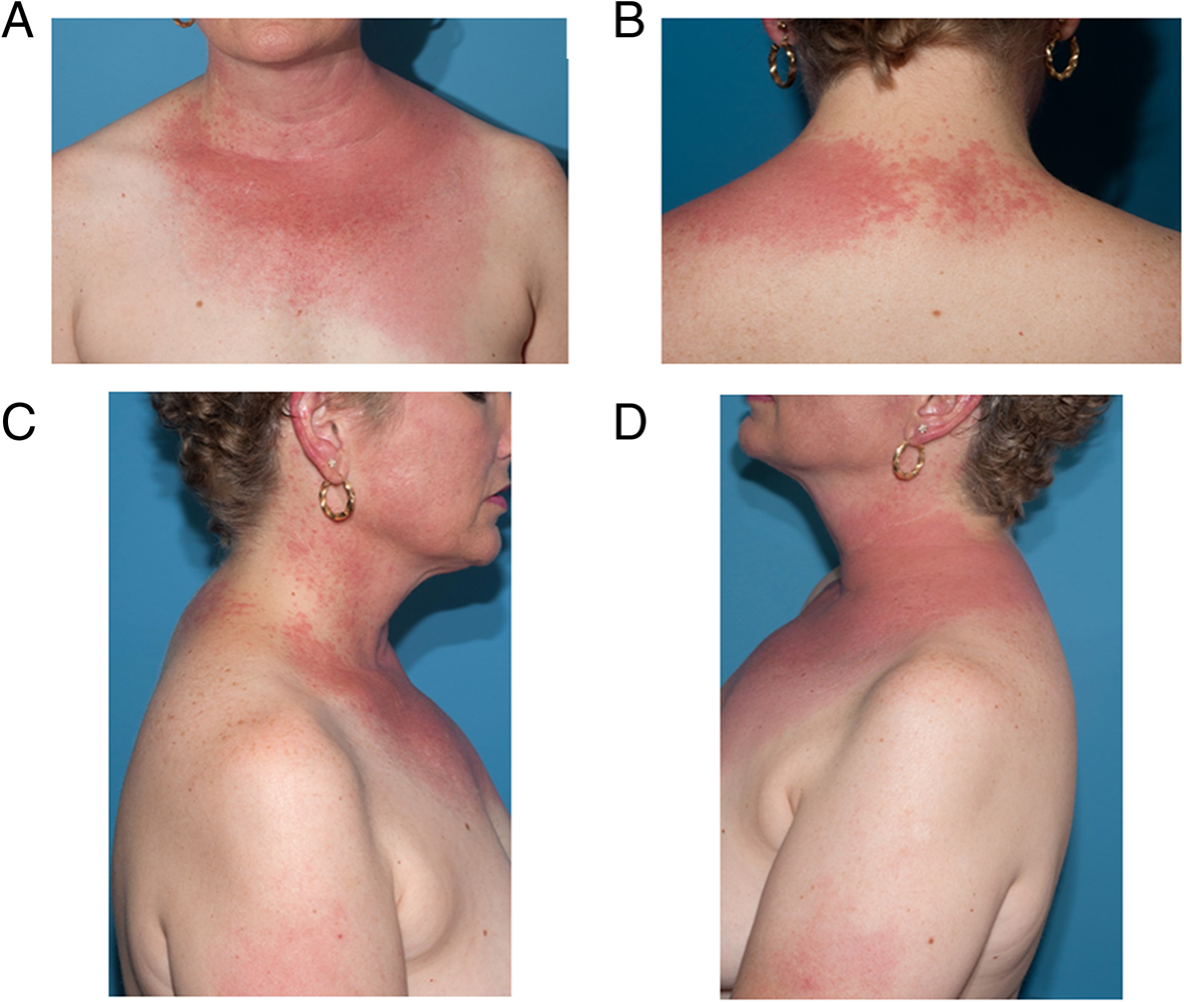
**Dermatologic adverse events to vandetanib. A)** Anterior chest. **B)** Upper portion of the back of the neck. **C** and **D)** Shoulders and arms showing sparing non-sun exposed areas.

A 51-year-old female with poorly differentiated, metastatic follicular thyroid carcinoma presented with a cutaneous eruption that developed over 3 to 4 days. One month prior to the development of the rash, she had begun therapy on a clinical trial with a combination of vandetanib at 300 mg by mouth daily and everolimus, a mammalian target of rapamycin (mTOR) inhibitor, at 5 mg by mouth daily (NCT01582191). She had a five-year history of thyroid cancer which had progressed despite thyroidectomy, radioactive iodine ablation therapy, chemotherapy, targeted therapy, radiation, and other novel agents. During follow up, her major complaint was new rash. The erythematous eczematous plaques started on the chest and posterior neck, with vesiculation of the posterior neck plaques one day after the rash was first noted. The lesions subsequently spread diffusely in sun-exposed areas over the chest, the upper portion of the back of the neck, and the bilateral forearms, sparing the shoulders, abdomen, pelvis, and legs. Borders were well-demarcated adjacent to sun-protected areas (Figure 1). She described the rash as pruritic with desquamation. The patient denied pain or involvement of the mucous membranes. She reported heavy sun exposure approximately 2 weeks prior to the visit, but did use SPF 50 sunscreen and wore long sleeves and long pants. However, she had, since that episode of heavy sun exposure, daily sun exposure without use of sunscreen. She did not report any new medications or changes to her current regimen. She held the study drugs for 1 day prior to the visit but otherwise was 100% compliant over the past month. Due to Grade 3 skin rash, the patient stopped the vandetanib and everolimus after being seen in clinic.

The patient was prescribed a 4 mg methylprednisolone dose pack, hydroxyzine for itching, clobetasol shampoo, triamcinolone 0.1% cream and an antibiotic to prevent superinfection. Aggressive photoprotection was also recommended.

**Figure 2:**
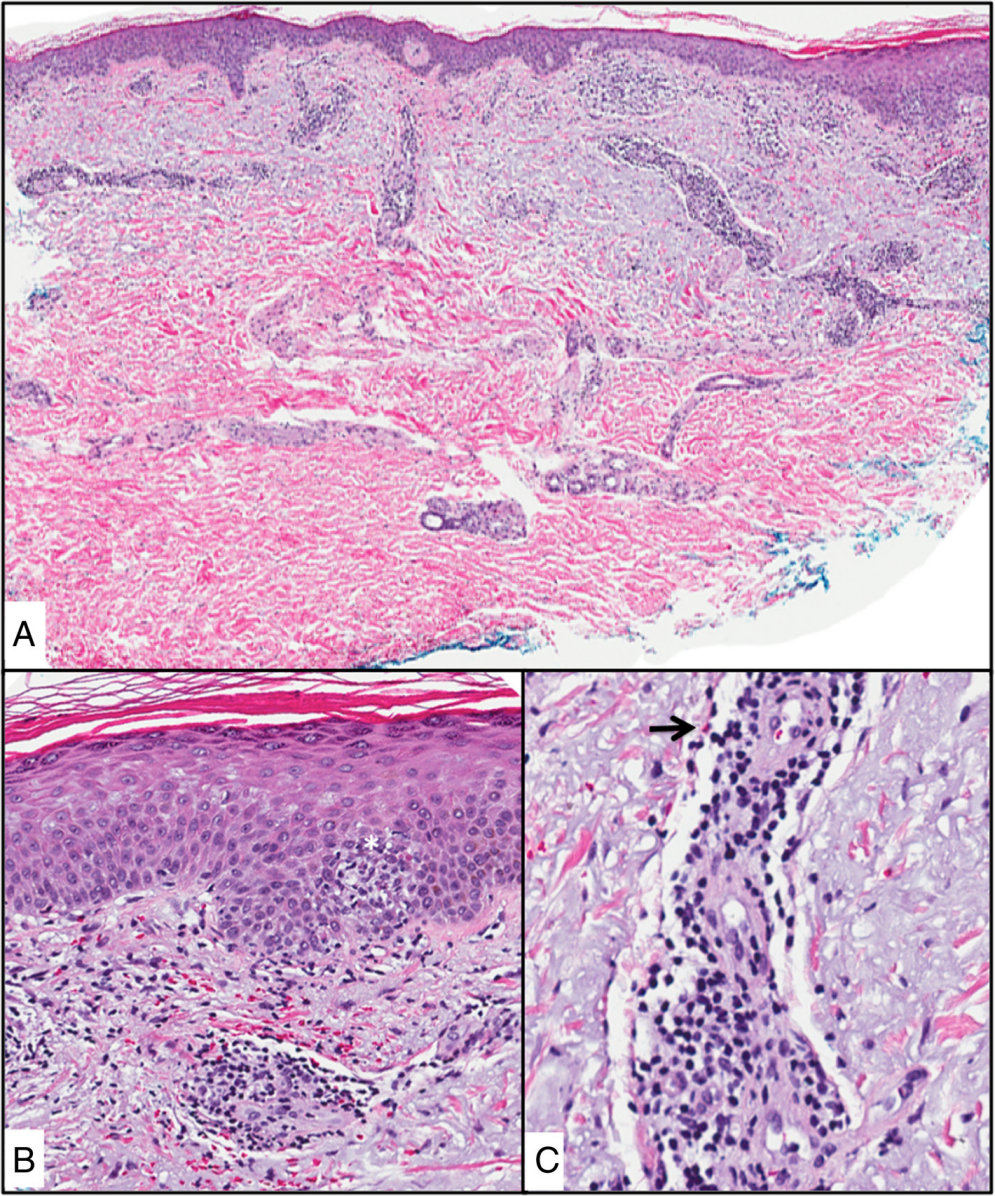
**Hematoxolin and eosin (H&E) A)** skin punch with superficial and deep perivascular lymphocytic infiltrate and epidermal spongiosis, **B)** epidermal spongiosis with exocytosis of lymphocytes (*) **C)** perivascular lymphocytes with eosinophils (arrow).

Subsequent dermatology evaluation revealed post-inflammatory erythema with few areas of eczematous dermatitis remaining. Photoallergic dermatitis was suspected. A 4 mm punch biopsy showed superficial perivascular dermatitis with eosinophils and focal spongiosis. Histologic features were consistent with a reaction to an internal antigen, such as a medication leading to photo allergic reaction (Figure 2). Based on the timing of the rash 2 weeks after the initial severe sun exposure, the photodistribution of the rash, history of vesiculation and pruritus, and the histologic features, the patient was diagnosed with photoallergic dermatitis. Laboratory results included a normal complete blood count and comprehensive metabolic panel.

Dermatology placed the patient on an oral prednisone taper. Vandetanib continued to be held. After an additional week, the patient noted decreased erythema and no further blistering. There were no new areas of involvement, but she continued to have rare eczematous plaques that may have been post inflammatory erythema.

The patient was re-challenged with vandetanib two weeks after resolution of the rash after completion of the steroid taper and with institution of strict photoprotection. The rash did not return and the patient is tolerating the study drug well. She continues to follow-up with the phase 1 clinic.

## Conclusions

Tyrosine kinase inhibitors, with various therapeutic targets, have come to the forefront of oncologic therapy in recent years. With block buster drugs such as imatinib for chronic myelogenous leukemia and gastrointestinal stromal tumor and vemurafenib for melanoma, drug companies and academic centers have formed collaborations to develop these types of agents in multiple tumor types. Vandetanib, an oral tyrosine kinase inhibitor, targets vascular endothelial growth factor receptor 2, the epidermal growth factor receptor, and RET [1,2]. It is approved by the United States Food and Drug Administration and European Medicines Agency for the treatment of advanced medullary thyroid cancer. Most recently, trials with vandetanib have also been conducted in hepatocellular carcinoma, and non-small cell lung cancer [3].

Tyrosine kinase inhibitors are known to have side effects including nontrivial dermatologic toxicity, usually manifesting as acneiform eruption, xerosis, eczema and cutaneous epithelial proliferations (e.g. actinic keratosis, keratoacanthoma, squamous cell carcinomas), with grade 1–2 rashes reportedly occurring in 11-82% of patients [4]. Photodistributed reactions rarely occur and can be divided into phototoxic or photoallergic etiologies [5]. Phototoxic reactions occur because of the damaging effects of light-activated compounds on cell membranes and, in some instances, DNA [5,6]. Photoallergic reactions are cell-mediated immune responses to a light-activated compound [5]. Phototoxic reactions develop in most individuals if they are exposed to sufficient amounts of light and drug, and develop within days of ultraviolet (UV) exposure [5]. Photoallergic reactions usually take days to weeks to develop as the immune response develops [5]. Phototoxic reactions occur more commonly than photoallergic reactions. In our case, an eruption appeared 2 weeks after the intense sun exposure, the lesions were pruritic, and limited to sun exposed areas therefore based on clinical presentation was more likely a photoallergic rather than phototoxic rash.

In the literature, few cases of photosensitivity reaction to vandetanib have been described in detail. However, it is important to note that in clinical trials with vandetanib, rash is commonly listed as a side effect although without specific detail given [7-9]. In one trial, up to grade 2 rash developed in 12 of 46 (26%) patients using vandetanib for metastatic breast cancer [8]. In other cases more severe reactions have been reported. For example, Chang et al. reports a case of a 60-year-old man with hepatocellular carcinoma treated on trial with vandetanib who developed bullous lesions related to sun exposure [10]. Kong et al. reports two cases of photo-toxicity induced hyperpigmentation [11]. Giacherro reports 21 of 63 (33%) patients developed erythematous skin eruptions ranging from exaggerated sunburn after moderate sun exposure to a severe photodistributed erythematous eruption associated with desquamation and pruritus [12]. These trials findings are summarized in Table 1.

**Table 1:** **Photosensitivity reactions to vandetanib reported in the literature**

**First author, year (Ref.)**	**Trial design**	**Vandetanib arm**	**Control arm**	**Enrollment no.**	**Sample size**	**Underlying cancer**
Katsuyuki Kiura, 2008 [9]	Phase IIa	100-300 mg/day	N/A	53	12	NSCLC
Heidi Kong, 2009 [11]	N/A	N/A	N/A	N/A	1	Recurrent brain tumor
Chih-Hsiang Chang, 2009 [10]	Phase II	300 mg/day	N/A	N/A	1	HCC
Damien Giacchero, 2012 [12]	Phase II/III	300 mg/day	Placebo	63	23	Metastatic thyroid cancer

Photosensitizing chemicals usually have a low molecular weight (200 to 500 Da), are planar, tricyclic, or polycyclic configurations and often contain heteroatoms that enable resonance stabilization [6,10]. They absorb UV light, a characteristic that is essential to be regarded as a photosensitizer. Vandetanib is a low–molecular weight molecule with a polycyclic structure [6,10]. Thus, it is plausible that vandetanib might be able to induce photosensitivity [6,10]. It also appears the degree of sun exposure does correlate with the severity of the rash [13].

In this patient, it is also important to consider photo accentuation of a typical drug eruption or other non-specific common polymorphous skin reactions found in many patients on experimental agents. In this case, however, the eruption was thought to be most consistent with a photoallergic reaction as the patient’s lesions were limited to photo-distributed areas (including sharp borders adjacent to sun-protected skin), the delayed onset of the eruption after the sun exposure and drug exposure, and the negative re-challenge when the patient was exercising strict photoprotection. The histology further supported a photoallergic versus phototoxic reaction as did the delayed onset of the eruption.

There are no defined guidelines as to the safety of re-challenge in patients who experience severe photo-induced reaction with vandetanib. It is been reported that these reactions usually resolve with the use of sunscreen and avoidance of sun exposure. In our case, holding the drug and giving both topical and oral steroids resulted in clearance of the rash. Ultimately, vandetanib was resumed with strict photoprotection and the patient tolerated continuance of therapy without further issues.

In conclusion, treatment with vandetanib and similar agents, either alone or in combination, may result in photodistributed rashes including photoallergic reactions. We hope to expand on the knowledge of skin reactions with the use of vandetanib to aid future researchers.

## Consent

Written informed consent was obtained from the patient for publication of this Case report and any accompanying images. A copy of the written consent is available for review by the Editor of this journal.

## Abbreviations

mTOR: Mammalian target of rapamycin
UV: Ultraviolet
